# P133: The practice of hand hygiene among manicures and pedicures in Brazil

**DOI:** 10.1186/2047-2994-2-S1-P133

**Published:** 2013-06-20

**Authors:** JL Garbaccio, AC Oliveira, AO de Paula

**Affiliations:** 1 Nursing, Federal University of Minas Gerais - Brazil, Belo Horizonte, Brazil

## Introduction

Hand hygiene is the primary mechanism to control the dissemination of microorganisms. Manicures and pedicures touch the hands and the feet of customers that can be very contaminated.

## Objectives

This study evaluated the practice of hand hygiene of manicures/pedicures in Brazil.

## Methods

This Survey included a random sample of 200 professionals older than 18, covering 200 beauty salons of Belo Horizonte, in Brazil. A questionnaire was used (Jul/12-Jan/13) to obtain information of demographics and their knowledge about the actual practice of hand washing. The results were analyzed by the statistical program SPSS. The study was approved by the Ethics Committee of the Federal University of Minas Gerais.

## Results

All professionals interviewed were women with an average age of 30, more than 10 years of experience (11%), working more than 8 hours per day (57%), less than a year in that salon (34%). As for education 55% had completed high school, 54% had done some training course in the field, yet 38% became manicure/pedicure by her own initiative. Moreover, 76% had never received any training in biosafety. From the knowledge questions, almost all of the respondents (99%) said it was important to wash their hands between customers schedules, using liquid soap stored in dispensers (81%) and dry them with a disposable paper towel (86%). However, 34% reported that they were not washing their hands, 14% using bar soap and 17% used a tissue towel without a daily change. The main reason for not joining the practice was the lack of time between appointments. There was no statistically significant association between demographics and the practice of hand washing (p>0.05). We evaluated the use of accessories like rings, bracelets and watches as well as the length of the nails of the professionals. More than half (53%) reported not removing the accessories, but on the day of the interview, 60% were found to be using at least one. The nails of 44% of the manicures/pedicures were long despite 38% stated that they keep them short.

## Conclusion

It was concluded that educational actions for manicures/pedicures should be implemented to encourage them to adhere to the hand washing practice that is so important for their own health and that of their customers’.

## Funding agent

Fundação de Amparo a Pesquisa de Minas Gerais. Programa Pesquisador Mineiro nº 00340-11

## Disclosure of interest

None declared

